# Serum oxidative stress biomarkers (MDA, SOD, GSH-PX) and IL-6/TNF-α ratio as biochemical indicators of disease progression in pediatric cariogenic pulpitis

**DOI:** 10.5937/jomb0-61223

**Published:** 2026-03-17

**Authors:** Mengxing Wang, Tian Xia, Ying Wang

**Affiliations:** 1 Capital Medical University, Capital Center for Children's Health, Department of Stomatology, China

**Keywords:** IL-6/TNF-α ratio, oxidative stress, malondialdehyde (MDA), superoxide dismutase (SOD), glutathione peroxidase (GSH-Px), pediatric cariogenic pulpitis, biochemical markers, odnos IL-6/TNF-α, oksidativni stres, malondialdehid (MDA), superoksid dismutaza (SOD), glutation peroksidaza (GSH-Px), pedijatrijski kariogeni pulpitis, biohemijski markeri

## Abstract

**Background:**

Cariogenic pulpitis is a common pediatric oral disease in which oxidative stress and inflammatory imbalance are increasingly recognized as important pathogenic mechanisms. Biochemical markers such as malondialdehyde (MDA), superoxide dismutase (SOD), glutathione peroxidase (GSH-Px), and the serum IL-6/TNF-<span style="color: rgb(32, 33, 34); font-family: sans-serif; font-size: 16px; background-color: rgb(248, 249, 250);"> α</span> ratio may provide insight into disease severity and progression.

**Methods:**

A total of 64 children with cariogenic pulpitis admitted between May 2023 and October 2024 were included, along with 64 healthy children as controls. Serum MDA, SOD, GSH-Px, and IL-6/TNF-<span style="color: rgb(32, 33, 34); font-family: sans-serif; font-size: 16px; background-color: rgb(248, 249, 250);"> α</span> ratio were measured using standard biochemical assays (TBA colorimetry, xanthine oxidase method, NADPH-coupled assay, and ELISA). Group comparisons were performed, and correlations with disease stage and caries depth were analyzed using Pearson correlation coefficients.

**Results:**

Compared with controls, children with pulpitis had significantly higher MDA and IL-6/TNF-<span style="color: rgb(32, 33, 34); font-family: sans-serif; font-size: 16px; background-color: rgb(248, 249, 250);"> α</span> levels (t = 18.542 and 20.639, both P &lt; 0.001) and lower SOD and GSH-Px activities (t = 8.279 and 12.449, both P &lt; 0.001). Within the pulpitis group (30 early stage, 24 middle stage, 10 late stage), MDA levels were positively correlated with both caries depth and disease severity (r = 0.82, P &lt; 0.05), while SOD (r = -0.78, P &lt; 0.05) and GSH-Px (r = -0.75, P &lt; 0.05) were negatively correlated. The IL-6/TNF-<span style="color: rgb(32, 33, 34); font-family: sans-serif; font-size: 16px; background-color: rgb(248, 249, 250);"> α</span> ratio was positively correlated with both stage and depth (r = 0.71, P &lt; 0.05), increasing significantly with lesion depth (r = 0.56, P &lt; 0.05).

**Conclusions:**

Elevated MDA and IL-6/TNF-<span style="color: rgb(32, 33, 34); font-family: sans-serif; font-size: 16px; background-color: rgb(248, 249, 250);"> α</span> ratio reflect greater oxidative and inflammatory burden and are associated with advanced stages of pediatric cariogenic pulpitis, whereas higher SOD and GSH-Px activities are associated with earlier disease. These biochemical indices may serve as adjunctive laboratory markers for evaluating disease progression and guiding therapeutic monitoring in pediatric pulpitis.

## Abstract (sr)

**Uvod:**

Kariogeni pulpitis je česta pedijatrijska oralna bolest kod koje se oksidativni stres i inflamatorni disbalans sve vise prepoznaju kao važni patogeni mehanizmi. Biohemijski markeri kao sto su malondialdehid (MDA), superoksid dismutaza (SOD), glutation peroksidaza (GSH-Px) i odnos IL-6/TNF-<span style="color: rgb(32, 33, 34); font-family: sans-serif; font-size: 16px; background-color: rgb(248, 249, 250);">α</span> u serumu mogu pružiti uvid u težinu i progresiju bolesti.

**Metode:**

Ukupno je uključeno 64 dece sa kariogenim pulpitisom primljenim između maja 2023. i oktobra 2024. godine, zajedno sa 64 zdrave dece kao kontrolne grupe. Serumski MDA, SOD, GSH-Px i odnos IL-6/TNF-<span style="color: rgb(32, 33, 34); font-family: sans-serif; font-size: 16px; background-color: rgb(248, 249, 250);">α</span> mereni su korišćenjem standardnih biohemijskih testova (TBA kolorimetrija, metoda ksantin oksidaze, NADPH-kuplovani test i ELISA). Izvrsena su poređenja grupa, a korelacije sa stadijumom bolesti i dubinom karijesa analizirane su korišćenjem Pirsonovih koeficijenata korelacije.

**Rezultati:**

U poređenju sa kontrolnom grupom, deca sa pulpitisom imala su značajno vise nivoe MDA i IL-6/TNF-<span style="color: rgb(32, 33, 34); font-family: sans-serif; font-size: 16px; background-color: rgb(248, 249, 250);">α</span> (t = 18,542 i 20,639, oba P &lt; 0,001) i niže aktivnosti SOD i GSH-Px (t = 8,279 i 12,449, oba P &lt; 0,001). Unutar grupe sa pulpitisom (30 ranog stadijuma, 24 srednjeg stadijuma, 10 kasnog stadijuma), nivoi MDA su bili pozitivno korelirani i sa dubinom karijesa i sa težinom bolesti (r = 0,82, P &lt; 0,05), dok su SOD (r = -0,78, P &lt; 0,05) i GSH-Px (r = -0,75, P &lt; 0,05) bili negativno korelirani. Odnos IL-6/TNF-a je bio pozitivno koreliran i sa stadijumom i sa dubinom (r = 0,71, P &lt; 0,05), značajno se povećavajući sa dubinom lezije (r = 0,56, P &lt; 0,05).

**Zaključak:**

Povišen odnos MDA i IL-6/TNF-<span style="color: rgb(32, 33, 34); font-family: sans-serif; font-size: 16px; background-color: rgb(248, 249, 250);">α</span> odražava većeoksidativno i inflamatorno opterećenje i povezan je sa uznapredovalim stadijumima pedijatrijskog kariogenog pulpitisa, dok su vece aktivnosti SOD i GSH-Px povezane saranijom bolešću. Ovi biohemijski indeksi mogu poslužiti kaododatni laboratorijski markeri za procenu progresije bolesti ivođenje terapijskog praćenja kod pedijatrijskog pulpitisa.

## Introduction

Cariogenic pulpitis is among the most common oral diseases in children and has a substantial impact on both oral health and quality of life [1][2]. In recent years, growing attention to its underlying mechanisms has highlighted the pivotal roles of oxidative stress and inflammatory responses in its pathogenesis [3].

Oxidative stress arises from an imbalance between pro-oxidant and antioxidant systems, leading to excessive generation of reactive oxygen species (ROS) and consequent cellular and tissue injury [4][5][6]. Malondialdehyde (MDA), a terminal product of lipid peroxidation, is a reliable biomarker of oxidative stress burden. In contrast, superoxide dismutase (SOD) and glutathione peroxidase (GSH-Px) are key antioxidant enzymes that mediate ROS detoxification [7]. Specifically, SOD catalyzes the dismutation of superoxide anions into hydrogen peroxide and molecular oxygen, while GSH-Px reduces hydrogen peroxide to water, thereby preserving the delicate oxidant-antioxidant equilibrium [8].

The inflammatory response also plays a crucial role in the onset and progression of cariogenic pulpitis. Interleukin-6 (IL-6) and tumor necrosis factor-α (TNF-α) are major pro-inflammatory cytokines that initiate and amplify inflammatory cascades [9][10]. The serum IL-6/TNF-α ratio has been proposed as a composite indicator reflecting systemic inflammatory status. Notably, oxidative stress and inflammation may act synergistically in cariogenic pulpitis: ROS activates inflammatory signaling pathways and enhances cytokine release, while inflammatory cytokines further exacerbate oxidative damage.

From a medical biochemistry perspective, these biomarkers offer a window into the molecular mechanisms of disease. MDA reflects lipid membrane peroxidation, SOD and GSH-Px represent antioxidant defense capacity, and the IL-6/TNF-α ratio integrates cytokine dynamics into a quantifiable biochemical index. Monitoring these markers not only deepens our understanding of the pathological progression of pulpitis but may also facilitate translational applications in laboratory diagnostics, risk stratification, and therapeutic monitoring. Importantly, serum-based biochemical indicators are non-invasive, reproducible, and suitable for pediatric populations, highlighting their clinical utility.

Although prior studies have suggested potential links between oxidative stress, cytokines, and pulpitis, the relationships among MDA, SOD, GSH-Px, and the IL-6/TNF-α ratio with disease progression in children remain insufficiently clarified. Therefore, the present study aimed to investigate these biochemical markers in children with cariogenic pulpitis, analyze their correlations with disease stage and caries depth, and provide evidence for their potential role in early diagnosis and intervention.

## Materials and methods

### Participants

Between May 2023 and October 2024, sixty-four children with a clinical diagnosis of cariogenic pulpitis were consecutively recruited from our hospital. During the same period, sixty-four healthy children undergoing routine oral examinations were enrolled as controls. The two groups were comparable at baseline, with no statistically significant differences in demographic or clinical characteristics (P > 0.05; Table 1).

**Table 1:** Baseline characteristics [x̄±s, n(%)].

Group	n	Gender		Disease course<br>(days)
		Male	Female	
Cariogenic	64	35	29	25.60±4.50
Control	64	37	27	25.48±4.57
*t/χ^2^*		0.127		0.150
*P*		0.722		0.881

### Inclusion and exclusion criteria

Eligible participants were required to be between 3 and 12 years of age and to have a diagnosis of cariogenic pulpitis confirmed by clinical and radiographic examinations. Only children who presented with typical clinical symptoms, such as spontaneous pain, nocturnal aggravation of pain, and poorly localized pain, and whose guardians provided written informed consent, were included. Children were excluded if they had received antibiotics, corticosteroids, or other medications that might affect inflammatory responses within the preceding month, if they were diagnosed with other oral diseases such as periodontitis, gingivitis, or oral mucosal disorders, or if they had neuropsychiatric conditions or developmental delay that prevented cooperation with the study. Participants with known allergies to any of the laboratory reagents used were also excluded.

### Laboratory measurements

Following an overnight fast, five milliliters of venous blood were collected from each child and placed in sterile centrifuge tubes. The samples were centrifuged at 3000 r/min for 20 minutes, and the serum was separated, aliquoted, and stored until analysis. Serum interleukin-6 (IL-6) and tumor necrosis factor-α (TNF-α) concentrations were determined using enzyme-linked immunosorbent assay (ELISA) kits, and the IL-6/TNF-α ratio was subsequently calculated. This method provided a sensitive and specific biochemical assessment of systemic inflammatory activity.

Malondialdehyde (MDA) levels were measured using the thiobarbituric acid (TBA) colorimetric assay. Serum samples were incubated with TBA reagent in acetic buffer at 95°C to generate a red MDA-TBA adduct, which was quantified spectrophotometrically at 532 nm. Superoxide dismutase (SOD) activity was determined by the xanthine-xanthine oxidase method, based on inhibition of pyrogallol autoxidation, with absorbance monitored at 420 nm. One unit of SOD activity was defined as the enzyme amount required to inhibit pyrogallol autoxidation by 50%. Glutathione peroxidase (GSH-Px) activity was assessed using an NADPH-coupled assay, wherein the decline in absorbance at 340 nm reflected NADPH consumption during peroxide reduction. Collectively, these assays yielded an integrated biochemical profile of oxidative stress and antioxidant defenses in the study population.

### Clinical evaluation

The severity of pulpitis was assessed by experienced pediatric dentists, who classified cases as early, middle, or late stage. Classification was based on a combination of clinical symptoms, including pain severity and frequency as well as the presence of spontaneous pain; oral examination findings, including caries depth and pulp exposure; and radiographic evaluation of carious extension and periapical involvement. Caries depth was recorded as an additional variable for analysis. Comparisons of serum MDA, SOD, GSH-Px, and IL-6/TNF-α ratio were performed between groups, and their associations with disease stage and caries depth were explored.

### Statistical analysis

All statistical analyses were conducted using Statistic Package for Social Science (SPSS) software, version 26.0 (IBM, Armonk, NY, USA). Categorical variables were summarized as counts and percentages and analyzed using the χ^2^ test, while continuous variables were expressed as mean ± standard deviation (SD) and compared using the independent-samples t test. Correlations between oxidative stress markers (MDA, SOD, GSH-Px) and the IL-6/TNF-α ratio with disease stage and caries depth were evaluated using Pearson correlation analysis. A P-value of less than 0.05 was considered to indicate statistical significance.

## Results

### Comparison of serum biomarkers between groups

Children with cariogenic pulpitis exhibited significantly higher serum levels of MDA and IL-6/TNF-α compared with healthy controls (t = 18.542 and 20.639, both P < 0.001). In contrast, the levels of SOD and GSH-Px were significantly lower in the pulpitis group (t = 8.279 and 12.449, both P < 0.001). These findings indicate that pulpitis is characterized by a biochemical profile of enhanced oxidative stress and systemic inflammation accompanied by reduced antioxidant defense (Table 2).

**Table 2:** Serum biomarkers in children with cariogenic pulpitis vs controls (mean±SD).

Group	n	MDA (nmol/mL)	SOD<br>(U/mL)	GSH-Px (U/mL)	IL-6/<br>TNF-α
Cariogenic pulpitis	64	2.49±0.43	85.27±8.62	42.54±4.55	4.11±0.89
Control	64	1.16±0.38	98.53±9.48	53.41±5.30	1.67±0.32
*t*		18.542	8.279	12.449	20.639
*P*		<0.001	<0.001	<0.001	<0.001

### Correlations of biomarkers with disease stage

Among the 64 children with cariogenic pulpitis, 30 were classified as early stage, 24 as middle stage, and 10 as late stage. Pearson correlation analyses demonstrated that MDA levels increased in parallel with caries depth and severity of pulpal inflammation, showing a strong positive correlation (r = 0.82, P < 0.05). Conversely, SOD and GSH-Px activities declined with disease progression, both displaying significant negative correlations with clinical stage and caries depth (r = -0.78 and -0.75, respectively, P < 0.05). The IL-6/TNF-α ratio also rose progressively across disease stages, with a positive correlation (r = 0.71, P < 0.05). These trends highlight the tight link between biochemical markers of oxidative stress, inflammatory imbalance, and clinical progression of pulpitis (Table 3).

**Table 3:** Biomarkers by disease stage (mean±SD).

Stage	n	Caries depth<br>(mm)	Pulpal<br>hyperemia	MDA<br>(nmol/mL)	SOD<br>(U/mL)	GSH-Px<br>(U/mL)	IL-6/TNF-α
Early	30	1.26±0.32	Mild	1.25±0.26	96.52±7.61	52.48±7.45	3.45±0.67
Middle	24	2.85±0.76	Moderate	1.92±0.32	88.46±6.60	45.43±5.48	4.89±1.12
Late	10	4.52±1.14	Severe	2.79±0.51	82.38±5.33	38.53±4.68	6.72±1.45

### Correlations of biomarkers with caries depth

When cases were stratified by lesion depth, 32 children had carious lesions ≤2.0 mm, 25 children had lesions measuring 2.0-4.0 mm, and 7 children had lesions 4.0 mm. Analysis revealed that the IL-6/TNF-α ratio increased markedly with deeper caries (r = 0.56, P < 0.05), indicating that progressive structural damage is accompanied by a measurable biochemical escalation of inflammatory burden (Table 4).

**Table 4:** Caries depth (x̄±s).

Depth category<br>(mm)	n	Caries depth (mm),<br>mean±SD
≤2.0	32	1.45±0.67
4.0	25	2.89±1.12
4.0	7	4.72±1.45

## Discussion

The pathogenesis of cariogenic pulpitis in children is multifactorial, with bacterial infection as the predominant etiological driver. Oral microorganisms— particularly Streptococcus mutans and Lactobacilli—form dental plaque on tooth surfaces, where they metabolize dietary carbohydrates into organic acids that demineralize enamel and initiate caries [11][12][13]. As lesions progress, bacterial byproducts and toxins infiltrate the dentin-pulp complex, eliciting a robust inflammatory response. Anatomic and histologic characteristics of primary and young permanent teeth further heighten vulnerability: enamel and dentin are thinner, less mineralized, and more permeable; the dentinal tubules are wider; and the pulp is comparatively loose, richly innervated, and highly vascularized, facilitating rapid spread of infection and inflammation. Behavioral and environmental factors also contribute: high sugar consumption, frequent snacking, and inadequate oral hygiene foster cariogenic biofilm formation. In addition, systemic conditions - such as impaired immune function or nutritional deficiencies - can increase susceptibility to infection and exacerbate pulpal inflammation [14][15].

Oxidative stress, defined as an imbalance between pro-oxidants and antioxidant defenses, is a key mechanism in this process [4][16]. Excessive production of reactive oxygen species (ROS) leads to lipid peroxidation, protein modification, and DNA damage. Malondialdehyde (MDA), a terminal lipid peroxidation product, is widely recognized as a reliable biochemical marker of oxidative stress. In this study, MDA levels increased progressively with the severity of pulpitis, suggesting that bacterial infection and the associated inflammatory cascade promote ROS accumulation and lipid peroxidation [17][18][19]. Elevated MDA not only reflects tissue damage but may also exacerbate membrane dysfunction and signal transduction abnormalities, further aggravating pulpal injury.

Conversely, SOD and GSH-Px are key enzymatic antioxidants that form the frontline defense against ROS. The progressive decline in their activities with disease advancement likely reflects both excessive utilization under sustained oxidative stress and cytokine-driven suppression of antioxidant enzyme expression. This reduction in SOD and GSH-Px undermines cellular redox capacity, amplifies oxidative injury, and perpetuates a self-reinforcing cycle that accelerates pulpal pathology.

Cytokines also play pivotal roles in pulpitis. Interleukin-6 (IL-6) is a multifunctional cytokine involved in immune cell growth, differentiation, antibody production, and acute-phase responses. In cariogenic pulpitis, bacterial stimuli activate immune cells to secrete IL-6, which enhances leukocyte recruitment and stimulates osteoclastogenesis, contributing to alveolar bone resorption and worsening pulp disease [20][21]. TNF-α, primarily secreted by macrophages and T lymphocytes, exerts broad biological effects. It promotes leukocyte adhesion and migration, increases vascular permeability, and amplifies the release of inflammatory mediators. Excessive TNF-α secretion leads to edema, hyperemia, and pulpal pain, and may trigger apoptosis, resulting in further structural and functional damage.

Oxidative stress and inflammation are not independent processes but reinforce each other. ROS activate signaling cascades such as the NF-kB pathway, promoting degradation of its inhibitor IkB and facilitating transcription of I L-6 and TNF-α genes. These cytokines in turn stimulate further ROS production and suppress antioxidant enzyme activity, perpetuating oxidative imbalance. This bidirectional crosstalk creates a self-sustaining cycle of oxidative damage and inflammation that underlies the progression of cariogenic pulpitis.

Our results confirmed these interactions, showing that MDA and the IL-6/TNF-α ratio increased significantly with disease severity, whereas SOD and GSH-Px declined. In early-stage pulpitis, lower MDA levels, higher antioxidant enzyme activity, and a relatively balanced IL-6/TNF-α ratio suggest a mild inflammatory and oxidative state. As disease advanced to middle and late stages, MDA and the IL-6/TNF-α ratio rose sharply, reflecting both intensified oxidative injury and cytokine imbalance. Importantly, the IL-6/TNF-α ratio appeared to capture the dynamic balance of inflammatory signaling, serving as an integrative biochemical index that parallels clinical progression.

From a clinical and biochemical perspective, monitoring the serum IL-6/TNF-α ratio alongside oxidative stress biomarkers may have practical value. A declining ratio after treatment could indicate effective control of inflammation and restoration of tissue homeostasis, whereas persistently high values may suggest poor therapeutic response and the need for treatment modification. Moreover, targeted modulation of I L-6 and TNF-α signaling may represent a promising therapeutic strategy for pediatric pulpitis, aiming to attenuate inflammatory damage while preserving or regenerating pulp vitality.

Despite these insights, this study has limitations. The sample size was relatively small, which may limit generalizability, and only serum biomarkers were analyzed. Local cytokine expression within pulp tissue may differ from systemic levels, and future studies should evaluate both local and systemic markers to provide a more comprehensive picture of the biochemical mechanisms underlying pulpitis. Expanding cohort size and incorporating additional oxidative and inflammatory biomarkers would further strengthen the evidence base.

In summary, this study demonstrates that elevated MDA and IL-6/TNF-α ratios are positively correlated with advanced stages and greater caries depth in pediatric cariogenic pulpitis, while decreased SOD and GSH-Px levels are associated with earlier stages and shallower lesions. These findings highlight the interplay of oxidative stress and inflammatory imbalance in pulpitis and underscore the potential of biochemical markers as adjunctive tools for diagnosis, disease monitoring, and therapeutic evaluation.

## Dodatak

### Financial disclosure

The authors declared that this study has received no financial support.

### Conflict of interest statement

All the authors declare that they have no conflict of interest in this work.
